# Early Alpha-Fetoprotein Response Predicts Sustained Tumor Response Following Immune Checkpoint Inhibitors Combined with Targeted Therapy in Liver Cancer

**DOI:** 10.3390/biomedicines12122769

**Published:** 2024-12-05

**Authors:** Jiahe Tian, Shida Pan, Yilin Wang, Yingying Yu, Siyu Wang, Yingjuan Shen, Luo Yang, Xiaomeng Liu, Qin Qiu, Junqing Luan, Fusheng Wang, Fanping Meng

**Affiliations:** 1 Peking University 302 Clinical Medical School, Beijing 100191, China; tianjh9888@163.com; 2 Department of Infectious Diseases, The Fifth Medical Centre of Chinese PLA General Hospital, National Clinical Research Centre for Infectious Diseases, Beijing 100039, China; wangsiyu_1982521@163.com (S.W.); 18501161267@163.com (Y.S.); xyinbj302@163.com (L.Y.); liuxiaomeng224@163.com (X.L.); qiuqinwangqiu@163.com (Q.Q.); ljq94630@163.com (J.L.); 3 Beijing Ditan Hospital, Capital Medical University, Beijing 100015, China; posda_pg@163.com; 4 Medical School of Chinese PLA, Beijing 100853, China; 83526542@163.com; 5 The First Affiliated Hospital of USTC, Division of Life Sciences and Medicine, University of Science and Technology of China, Hefei 230001, China; yuyingying1020@163.com

**Keywords:** liver cancer, hepatocellular carcinoma (HCC), immune checkpoint inhibitors (ICI), alpha-fetoprotein (AFP), biomarker, progression-free survival (PFS)

## Abstract

**Background:** Although immune checkpoint inhibitors (ICI) have revolutionized liver cancer treatment, some patients experience early tumor progression after therapy, missing the window for other potential treatments, such as neoadjuvant therapy. Therefore, identifying the predictive factors for early progression is critical for timely therapeutic adjustment and the optimization of patient outcomes. **Methods:** This retrospective study enrolled patients with liver cancer who received their first ICI combined with targeted therapy at the Fifth Medical Center of the PLA General Hospital between June 2022 and December 2023. Early tumor progression was defined as tumor progression within 6 months of therapy initiation. Multivariate logistic regression analysis was used to identify independent risk factors for early tumor progression, and overall survival (OS) curves were generated using the Kaplan–Meier method. **Results:** A total of 159 patients were enrolled. Multivariate logistic regression analysis indicated that patients with an early alpha-fetoprotein (AFP) response had a significantly reduced risk of early tumor progression (OR = 0.34, 95% CI: 0.13–0.84, *p* = 0.019), suggesting that an early AFP response is a protective factor against early progression. The area under curve (AUC) for the predictive model was 0.73 (95% CI: 0.63–0.83, *p* < 0.001). Stratified survival analysis showed that the median overall survival (mOS) in the early AFP response group was significantly longer than that in the poor response group (17.3 months vs. 6.1 months, HR = 2.11, 95% CI: 1.19–2.74, *p* = 0.009). **Conclusions:** Early AFP response is not only an effective biomarker for identifying high-risk patients prone to early tumor progression but is also significantly associated with long-term survival in liver cancer patients treated with ICI combined with targeted therapy. This finding will enable clinicians to make timely therapeutic adjustments and optimize treatment outcomes, thereby improving both progression-free survival and overall survival.

## 1. Introduction

Liver cancer, particularly hepatocellular carcinoma (HCC), is the sixth most common cancer and the third leading cause of cancer-related mortality worldwide, after lung and colorectal cancer [1]. In recent years, the application of immune checkpoint inhibitors (ICI) combined with targeted therapy has significantly changed the treatment landscape for HCC, resulting in extended progression-free (PFS) and overall survival (OS) of patients [2,3]. Notably, following the success of the IMbrave150 trial, ICI combined with targeted therapy has emerged as the new standard of care for the first-line treatment of advanced HCC [4,5]. However, despite the promise of this combination therapy, a substantial number of patients remain unresponsive or develop resistance, with some experiencing early tumor progression after treatment initiation, leading to poor prognoses [2,6,7].

Alpha-fetoprotein (AFP) is a glycoprotein with an immunoregulatory role in fetal development [8,9]. Although expressed at minimal levels in adults, elevated AFP levels can indicate certain pathological conditions involving liver regeneration, hepatitis, or cancer [5,10]. Studies have established that both baseline AFP levels and post-treatment decline in serum AFP are associated with tumor response in patients receiving various systemic therapies, such as anti-angiogenic therapy and resection [11,12,13,14]. However, the specific relationship between early AFP response and early tumor progression following ICI combined with targeted therapy has not been thoroughly investigated. This gap highlights the need to explore how an early AFP response correlates with the efficacy of such combination treatments. Given the well-known role of AFP in reflecting the tumor burden and biological behavior in liver cancer, we hypothesized that an early AFP response could indicate favorable tumor dynamics in response to therapy, whereas a lack of response may suggest the need for treatment adjustments. Therefore, investigating the relationship between early AFP response and early tumor progression is essential for optimizing patient management and improving the efficacy of ICI in combination with targeted therapy. This could potentially allow for the earlier identification of patients who may benefit from alternative therapeutic strategies, leading to more personalized and effective treatment plans.

This study aimed to conduct a retrospective analysis of clinical data from patients with liver cancer who received ICI combined with targeted therapy for the first time, exploring the predictive factors associated with early tumor progression to provide a reference for clinical decision-making.

## 2. Materials and Methods

### 2.1. Participants

**Figure 1:**
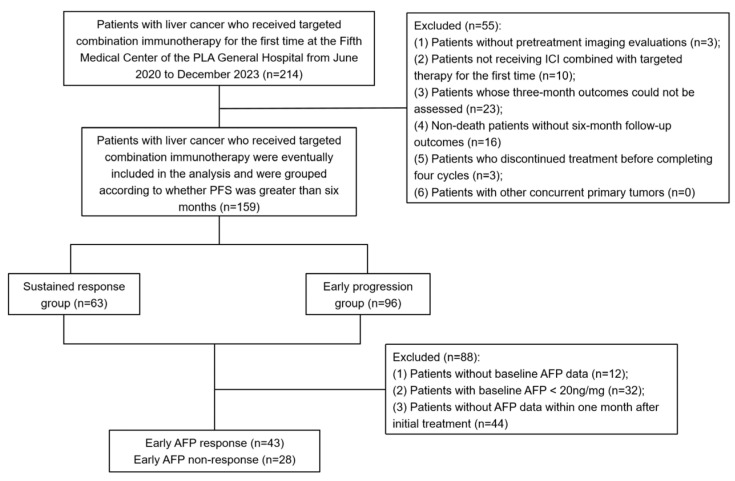
Flowchart.

A total of 214 patients with liver cancer who received ICI combined with targeted therapy at the Fifth Medical Center of the PLA General Hospital between June 2022 and December 2023 were included in this study. A retrospective analysis was conducted on the following patient information: medical history, laboratory test results, imaging findings, and treatment history. The follow-up period was extended from the first ICI administration of immune checkpoint inhibitors until 1 June 2024. The inclusion criteria for the study were as follows: (1) a diagnosis of primary liver cancer confirmed by imaging (CT, MRI) or pathology, without any other concurrent primary tumors; (2) at least one measurable lesion according to the RECIST 1.1 criteria; (3) first-time treatment with ICI combined with targeted therapy; and (4) treatment with ICI combined with targeted therapy for at least four cycles, with clinical outcomes assessed for a minimum of 3 months. The exclusion criteria were as follows: (1) patients without pretreatment imaging evaluations; (2) patients not receiving ICI combined with targeted therapy for the first time; (3) patients whose three-month outcomes could not be assessed; (4) non-death patients without six-month follow-up outcomes; (5) patients who discontinued treatment before completing four cycles; and (6) patients with other concurrent primary tumors. Based on the inclusion and exclusion criteria, 159 patients were included in this study for subsequent analyses (Figure 1).

Demographic and clinical characteristics at baseline and at each follow-up point were retrospectively analyzed: age, sex, etiology, Hepatitis B virus (HBV) infection, Child-Pugh stage, Barcelona Clinic Liver Cancer (BCLC) stage, Albumin–Bilirubin (a grading system that combines the levels of albumin and bilirubin to comprehensively assess liver function, ALBI) grade, tumor size (cm), treatment-related adverse reactions, routine blood tests, blood biochemistry, thyroid function, lymphocyte subset count, C-reactive protein (CRP) level (mg/L), IL-6 level (pg/mL), AFP level (ng/mL), neutrophil–lymphocyte ratio (NLR), Eastern Cooperative Oncology Group Performance Status (ECOG PS), imaging studies, and any previous treatment.

### 2.2. Drug Administration

The PD-1 inhibitors: sintilimab (Innovent Biologics, Suzhou, China), camrelizumab (Hengrui Pharma, Lianyungang, China), and tislelizumab (BeiGene, Shanghai, China) at a fixed dose of 200 mg, and toripalimab (Top Alliance, Shanghai, China) at 240 mg were prescribed for a 3-week cycle. The PD-1 and CTLA-4 inhibitors, cadonilimab (Akesobio, Guangzhou, China), at 240 mg were prescribed for a 2-week cycle. Sorafenib (Bayer Schering Pharma, Berlin, Germany) was administered at 400 mg/day, lenvatinib (Eisai, Tokyo, Japan) at 8 to 12 mg/day according to body weight, and bevacizumab (Qilu Pharmaceutical, Jinan, China) at 7.5 mg/kg for 3 weeks.

### 2.3. Definitions in Research

A summary analysis of large clinical trial data from both domestic and international sources indicated that the median progression-free survival (mPFS) for liver cancer treated with ICI combined with targeted therapy is approximately 4–8 months, with a median overall survival (mOS) of approximately 20 months [15,16,17,18]. Therefore, in this study, early tumor progression was defined as a PFS of less than 6 months following the initial ICI combined with targeted therapy, whereas sustained response was defined as a PFS of greater than 6 months after the first ICI combined with targeted therapy.

Baseline AFP levels were collected from all patients, excluding those with normal baseline AFP levels (AFP < 20 ng/mL). Following the study by Shao et al., an early response was defined as a >20% decrease in serum AFP levels compared to pretreatment levels within the first 4 weeks of treatment [13]. If multiple AFP measurements were obtained within the first 4 weeks, the highest level was used for the calculation.

### 2.4. Statistical Analyses

Statistical analysis was performed on the baseline and follow-up data of all study subjects. Continuous variables were described using mean ± standard deviation (mean ± SD) for normally distributed data or interquartile range (IQR) for skewed data and analyzed using independent samples *t*-test or Mann–Whitney U test. Categorical variables were described using frequency (n) and percentage (%), with comparisons made using chi-square tests or Fisher’s exact test. Chi-square analysis, without strict requirements on data distribution, can effectively examine whether there are distribution differences of categorical variables in the baseline data between two groups of patients, as well as whether there is an association between the grouping variable and each categorical variable.

Subsequently, we conducted univariate logistic regression analysis. The reason for using univariate analysis was to preliminarily screen out variables that might have a potential impact on early tumor progression. By examining each variable independently, we could identify those that showed a significant association with the outcome at a single-variable level. In a complex clinical context such as ours, multiple factors do not act in isolation, but rather interact with each other in influencing the outcome of early tumor progression. Univariate analysis alone cannot account for these interactions and may provide a misleading picture of the true relationship between variables and the outcome. Multivariate analysis allows us to consider all these factors simultaneously and tease out the independent contributions of each variable while accounting for the potential confounding effects. By including all the variables that were significant in the univariate analysis or those with clinical relevance in the multivariate model, we could more accurately determine the independent factors associated with early tumor progression. The odds ratios (OR) and 95% confidence interval (95% CI) were calculated to assess the strength of each predictive factor. To evaluate the performance of the multivariate logistic regression model, receiver operating characteristic (ROC) curves were used. The area under the ROC curve (AUC) was computed to quantify the discriminative ability of the model, with AUC values ranging from 0.5 to 1, and values closer to 1 indicating better predictive performance. Sensitivity and specificity were assessed at various threshold levels, and the optimal cutoff value was selected to enhance the prediction effectiveness. Additionally, based on the results of multivariate logistic regression analysis, a nomogram was constructed to visualize the prediction model. The nomogram assigned scores to each independent risk factor, providing a comprehensive assessment of the individual patient’s risk for early tumor progression.

The predictive performance of the model was validated using calibration curves and the concordance index, and the C-index was used to evaluate its discriminative ability. Calibration curves were used to compare the predicted probabilities with the actual observed outcomes for consistency. Calibration of the model was further assessed using the Hosmer–Lemeshow (HL) test. Decision curve analysis (DCA) was used to evaluate the clinical utility of the nomogram and determine the net benefit at various threshold levels. Kaplan–Meier (KM) survival curves were plotted for overall survival (OS), and the differences in mOS among the different groups were compared using the log-rank test. Data analysis was performed using R 4.3.0 statistical software and IBM SPSS Statistics (version 25). All statistical tests were two-sided, and a *p*-value < 0.05 was considered statistically significant.

## 3. Results

### 3.1. Patient Characteristics

In total, 159 patients with liver cancer who received ICI combined with targeted therapy at the Fifth Medical Center of the PLA General Hospital between June 2022 and December 2023 were included for further analysis. The patients were divided into a sustained response group (n = 63) and an early progression group (n = 96) based on whether the PFS was greater than 6 months. The average age of the patients was 57 ± 10 years, with 138 males (86.8%). In terms of diagnosis, hepatitis B and hepatitis C virus-related HCC accounted for 81.0% of the sustained response group, alcoholic liver cancer accounted for 14.3%, and intrahepatic cholangiocarcinoma accounted for 4.8%. In the early progression group, 81.3% had virus-related HCC, 13.5% had alcoholic liver cancer, and 5.3% had intrahepatic cholangiocarcinoma. Apart from a higher proportion of patients in the early progression group with AFP ≥ 400 ng/mL (46.8% vs. 24.6%, *p* = 0.005), AST ≥ 40 U/L (77.1% vs. 54.0%, *p* = 0.002), and tumors ≥ 5 cm (67.4% vs. 45.9%, *p* = 0.008), no other demographic or clinical characteristics showed statistically significant differences between the two groups (Table 1).

### 3.2. Risk Factors of Early Progression in Patients with Liver Cancer

To identify the relationship between clinicopathological factors and the early progression of liver cancer in patients receiving ICI combined with targeted therapy, we conducted a logistic regression analysis on the clinical data of the cohort. Univariate logistic regression analysis revealed that age, ALBI grade 3, tumor size ≥ 5 cm, AFP ≥ 400 ng/mL, early AFP response, AST > 4 0 U/L, alkaline phosphatase > 150 U/L, γ-glutamyl > 50 U/L, and absolute counts of white blood cells and monocytes were significant factors influencing PFS in liver cancer patients post-treatment. Following the inclusion of variables with *p* < 0.05 from the univariate analysis in the multivariate analysis, we found that early AFP response (OR: 0.34; 95% CI: 0.13–0.84) was a significant predictor of continuous response and served as an independent protective factor for sustained response to ICI combined with targeted therapy (Table 2).

**Figure 2:**
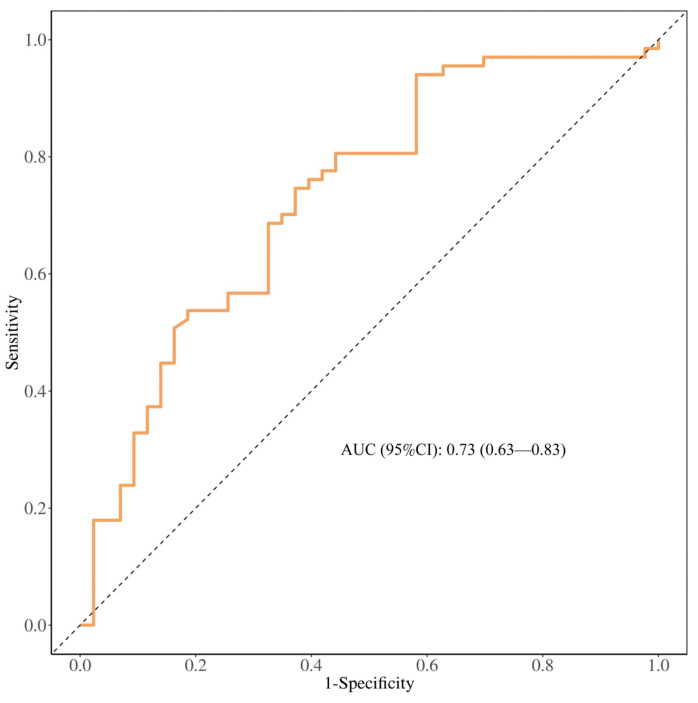
ROC curve analysis for the model. The *x*-axis denotes the false positive rate (1-specificity), and the *y*-axis represents the true positive rate (sensitivity). The area under the ROC curve (AUC) was calculated to be 0.73, signifying satisfactory discriminatory power.

Subsequently, the predictive performance of the multivariate logistic regression model was assessed using the ROC curve, which yielded an AUC of 0.73 (95% CI: 0.63–0.83), indicating good predictive accuracy (Figure 2).

**Figure 3:**
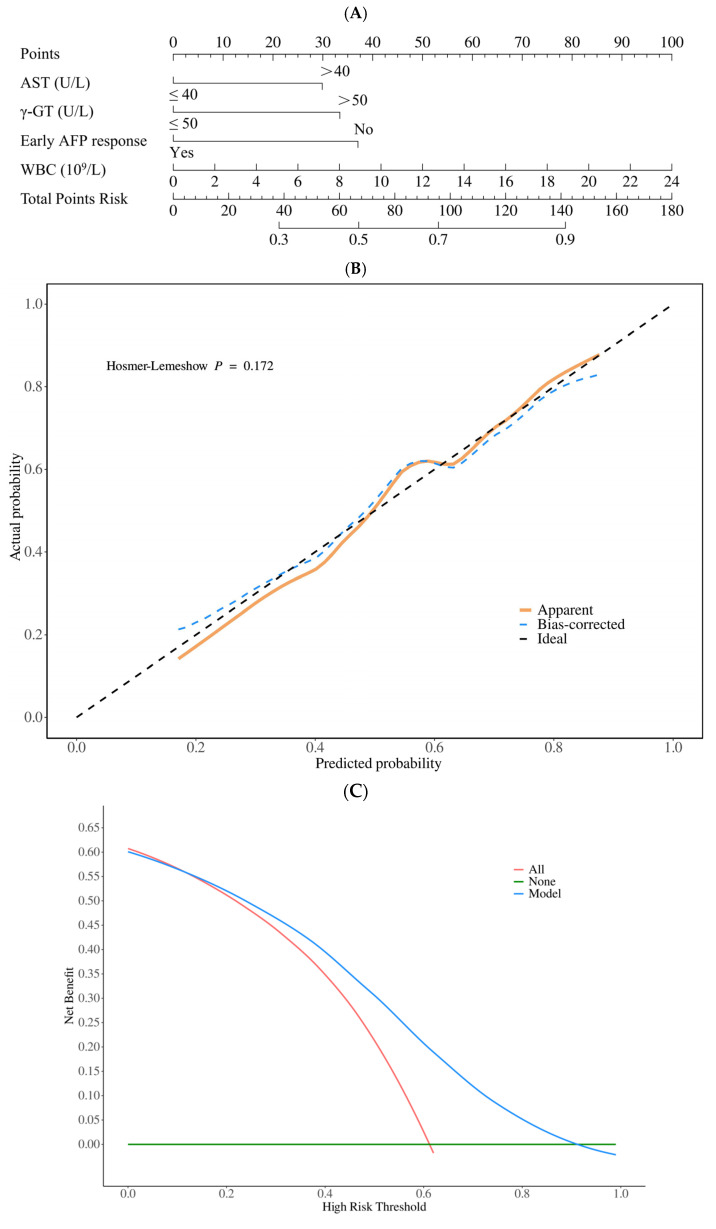
(**A**) Nomogram for predicting early progression of liver cancer patients. Each straight line in the figure represents different predictive variables, including AST > 40 U/L, γ-GT > 50 U/L, Early AFP response, and WBC. On each line, the corresponding score for each variable can be determined according to the relevant scale. By adding up the scores of each variable, a comprehensive score can be obtained on the total scoring axis, and the value on the survival probability axis below the corresponding comprehensive score is the predicted probability of early tumor progression. (**B**) Calibration curves to check the prediction accuracy of the model. (**C**) DCA was used to evaluate the clinical usefulness of the model.

Based on the results of the multivariate logistic regression analysis, we constructed a nomogram as a clinical prediction model. This model calculates the total score based on the individual values of the predictor variables and subsequently estimates the risk or survival probability for a particular event. From the distribution of the nomogram and the score lines for each variable, it is evident that AST levels greater than 40 U/L, γ-glutamyl transferase levels exceeding 50 U/L, poor early AFP response, and higher white blood cell (WBC) counts are associated with an increased risk of early progression following treatment (Figure 3A). We then validated the model’s predictive accuracy using a calibration curve, which demonstrated good agreement between predicted values and actual observations (*p* = 0.172) (Figure 3B). DCA was conducted to evaluate the clinical utility of the model, revealing a high net benefit within clinically relevant threshold ranges, confirming its effectiveness for clinical application (Figure 3C).

### 3.3. Early AFP Response Is Associated with Long-Term Survival Prognosis

**Figure 4:**
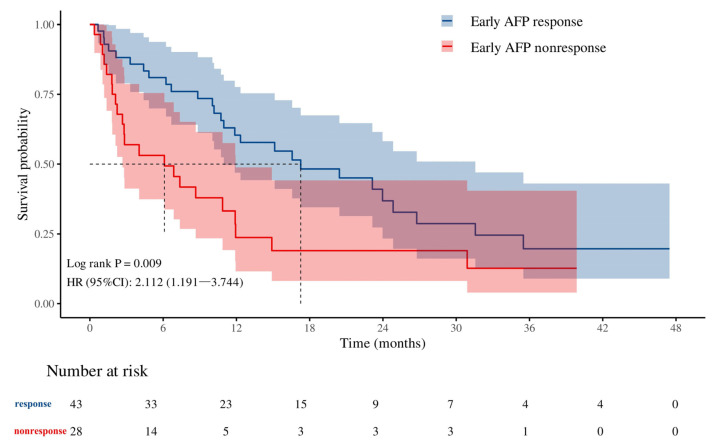
Association between early AFP response and mOS in liver cancer patients treated with ICI combined with targeted therapy. The survival probability of the Early AFP response group (blue curve) is higher than that of the Early AFP non-response group (red curve) at all time points.

These results indicate that an early AFP response serves as a protective factor against early tumor progression in patients with liver cancer following ICI combined with targeted therapy. Furthermore, we assessed the correlation between the early AFP response and OS. Kaplan–Meier survival analysis revealed that the mOS of the early AFP responder group was significantly longer than that of the poor responder group (17.3 months vs. 6.1 months, *p* = 0.009) (Figure 4). This finding suggests that a favorable early AFP response is not only associated with a reduced risk of early tumor progression but is also closely linked to long-term survival benefits for patients. Clinically, an early AFP response can serve as a valuable biomarker for evaluating the efficacy of ICI combined with targeted therapy, providing a basis for optimizing personalized treatment strategies and ultimately improving patient prognosis.

### 3.4. Relationship Between Early AFP Response and Early Tumor Response

Next, we explored the association between early AFP response and tumor response outcomes. We defined the tumor response at 3 months post-treatment as an early tumor response. Chi-square analysis of the early AFP response and early objective response rates revealed that the objective response rate (an indicator for evaluating the efficacy of cancer-treatment drugs, representing the proportion of patients whose tumors shrink to a certain standard after treatment, ORR) for early AFP responders was 39.5%, whereas for those with poor early AFP response, the ORR was only 10.1%, with a statistically significant difference (*p* = 0.014). The DCR demonstrated similar results (DCR = 74.4% vs. 32.1%, *p* = 0.001) (Table 3). These findings suggest that an early AFP response is closely linked to improved tumor response and may serve as a predictive marker for early treatment efficacy.

## 4. Discussion

This study revealed significant associations between various factors, including ALBI grade, baseline AFP levels, early AFP response, ALP, γ-glutamyl, AST, and absolute counts of leukocytes and monocytes, with early tumor progression in liver cancer patients following ICI combined with targeted therapy. Elevated levels of biochemical markers, such as AST and ALP, are associated with an increased risk of tumor progression. Changes in these markers may reflect liver dysfunction and enhanced tumor activity, suggesting that clinicians should pay close attention to these biochemical parameters when assessing patient prognosis. The increase in the monocyte count may be related to the body’s immune response, indicating the potential protective role of the immune system in tumor progression. Through multivariate analysis, we found that early AFP response was an independent protective factor against early progression, suggesting its potential as an important biomarker for evaluating treatment response. Kaplan–Meier (KM) curve analysis indicated that a favorable early AFP response is not only associated with a lower risk of early tumor progression but is also closely linked to long-term survival benefits for patients. Additionally, we identified a significant relationship between early AFP response and early (3 months post-treatment) objective tumor response. Chi-square test results showed a significant correlation between early AFP response and treatment response (*p* = 0.001), indicating that early AFP response may serve as an effective predictor of treatment response. Approximately 70% of patients with HCC express and secrete the glycoprotein AFP, which is widely regarded as being associated with the biological behavior of liver cancer [19,20]. Therefore, AFP is a conventional diagnostic biomarker for HCC in clinical practice and a potential target for immunotherapy [21,22,23]. Elevated AFP levels are typically correlated with higher tumor burden and poorer prognosis [24]. In our study, AFP levels (≥400 ng/mL) were significantly higher in the early progression group compared to the sustained response group, consistent with previous findings [25].

Neoadjuvant immunotherapy has been shown to effectively activate tumor immune responses, shrink tumors, and eliminate metastatic lesions [26]. Its clinical benefits have been demonstrated in various solid tumors, including triple-negative breast cancer, melanoma, non-small-cell lung cancer, and bladder cancer [27,28,29,30]. In liver cancer, the goals of neoadjuvant therapy are to treat micrometastases early, shrink the primary tumor to render it resectable, and improve PFS and OS [31]. This single-center, open-label, phase II clinical trial explored the efficacy of neoadjuvant cemiplimab (a PD-1 monoclonal antibody) in resectable hepatocellular carcinoma. The trial enrolled 21 patients with stage Ib, II, and IIIa HCC confirmed by imaging or biopsy, of which 20 underwent surgical resection. The results showed that 3 out of 20 patients (15%) achieved complete tumor necrosis, 7 out of 20 patients (35%) had significant tumor necrosis, and 13 out of 20 patients (65%) had less than 50% tumor necrosis. The treatment was also associated with a favorable safety profile [27]. However, neoadjuvant therapy has some potential risks. Disease progression may occur in patients who do not respond to treatment, potentially resulting in the loss of opportunity for curative surgery [31]. Additionally, severe treatment-related adverse events may affect the feasibility of surgery or increase surgical risk. In previous clinical trials, imaging assessments were typically conducted every 6–8 weeks, whereas in clinical practice, imaging is often performed approximately every 3 months or even less frequently [32]. Therefore, it is important to explore early predictive indicators for tumor progression, which can aid clinicians in the early identification of patients who may have a poor response to ICI combined with targeted therapy, allowing for timely adjustment to treatment plans. Several retrospective studies have demonstrated that baseline serum AFP levels and early on-treatment response of AFP are associated with therapeutic efficacy and prognosis in patients with HCC treated with ICI-based regimens [33,34,35,36]. In addition, a study showed that the AFP response, namely, the change in AFP 1 week after resection, is a simple and novel indicator of the oncologic effect of surgical treatment of HCC, predicts survival of patients and tumor recurrence independently of tumor-related risk factors, and can be obtained shortly after resection [14]. Currently, the combination of immunotherapy and targeted therapy has become the recommended first-line standard treatment regimen for liver cancer. Previous studies mostly only focused on the prediction of ICI treatment and did not consider the impact of targeted therapy, thus making it difficult to meet the current clinical practice requirements. In addition, previous studies were retrospective analyses of subjects who had participated in previous clinical trials, and there was a selection bias. Building on these prior findings, our study investigated the relationship between early AFP response and early tumor progression in patients undergoing ICI combined with targeted therapy. Our findings indicate that patients with a favorable early AFP response have a significantly reduced risk of tumor progression and a notably extended overall survival, suggesting that they may be better candidates for neoadjuvant therapy.

In our study, a significant gender disparity was observed, with the number of male participants being considerably greater than that of female participants. This finding is in line with the well-established epidemiological trends of liver cancer, both domestically and internationally. Understanding these gender differences in the prevalence of liver cancer is of crucial importance for formulating more targeted prevention and treatment strategies. Future research should further explore the biological and social determinants underlying such gender differences to improve the management and treatment outcomes of liver cancer.

Our study is the first to explore the relationship between early AFP response and early tumor progression based on real-world data. Although this study provides preliminary insights into the predictive factors for early progression, it has some limitations. Firstly, being a single-center study restricts the diversity of the patient population. The patients included predominantly represent an institutional cohort, lacking the heterogeneity that would be present in a multicenter study. Secondly, the relatively small sample size further compounds the issue. With a limited number of participants, the statistical power of the study is compromised. It becomes more challenging to detect subtle yet potentially important relationships between variables. Minor but significant predictors of early tumor progression might have been overlooked due to the insufficient sample size. Thirdly, regarding the analysis of AFP, while it has shown promise as a potential predictor of early tumor progression, there are also limitations. AFP levels can be influenced by various factors other than tumor progression, such as liver function status, presence of other liver diseases, or even individual physiological variations. This means that relying solely on AFP as a predictive marker may introduce false positives or false negatives in the assessment. Looking ahead, future prospective and multicenter studies are of utmost importance. These studies will not only expand the sample size but also incorporate a more diverse patient population, enhancing the generalizability of the findings. In particular, future research should focus on the joint analysis of multiple biomarkers. By combining AFP with other relevant biochemical markers, such as circulating tumor cells, tumor markers other than AFP (e.g., carcinoembryonic antigen, CA19-9), and specific gene expression profiles related to liver cancer, a more comprehensive and accurate predictive model can be developed. This approach will help to account for the individual variability in patient responses and the complex interplay between different factors influencing tumor progression. Through such comprehensive biomarker analysis, the reliability of the results and the predictive performance of the model can be significantly enhanced, providing a more solid basis for the development of personalized treatment strategies. Moreover, further exploration of the biological mechanisms underlying these factors will deepen our understanding of how ICI combined with targeted therapy affects tumor progression, enabling more effective optimization of this treatment combination in liver cancer patients

## Figures and Tables

**Table 1:** Baseline characteristics of liver cancer patients.

Variables	Total (n = 159)	Sustained Response Group (n = 63)	Early Progression Group (n = 96)	*p* Value
Sex, n (%)				0.266
Female	21 (13.2)	6 (9.5)	15 (15.6)	
Male	138 (86.8)	57 (90.5)	81 (84.4)	
Age, n (%)				0.274
≤60 y	104 (65.4)	38 (60.3)	66 (68.8)	
>60 y	55 (34.6)	25 (39.7)	30 (31.3)	
Diagnose, n (%)				1.000
Alcoholic HCC	22 (13.8)	9 (14.3)	13 (13.5)	
Hepatitis virus-associated HCC	129 (81.1)	51 (81.0)	78 (81.3)	
Cholangiocarcinoma	8 (5.0)	3 (4.8)	5 (5.2)	
TNM, n (%)				0.603
3	5 (26.3)	1 (14.3)	4 (33.3)	
4	14 (73.7)	6 (85.7)	8 (66.7)	
BCLC stage, n (%)				0.235
A	11 (7.8)	7 (12.3)	4 (4.8)	
B	29 (20.6)	13 (22.8)	16 (19.1)	
C (PVTT)	55 (39.0)	23 (40.4)	32 (38.1)	
C (M)	43 (30.5)	14 (24.6)	29 (34.5)	
D	3 (2.1)	0 (0.0)	3 (3.6)	
Child-pugh stage, n (%)				0.160
A	71 (44.7)	33 (52.4)	38 (39.6)	
B	81 (50.9)	29 (46.0)	52 (54.2)	
C	7 (4.4)	1 (1.6)	6 (6.2)	
ALBI grade, n (%)				0.112
1	13 (8.2)	8 (12.7)	5 (5.2)	
2	97 (61.1)	40 (63.5)	57 (59.4)	
3	49 (30.8)	15 (23.8)	34 (35.4)	
Tumor size, n (%)				0.008
<5 cm	63 (41.2)	33 (54.1)	30 (32.6)	
≥5 cm	90 (58.8)	28 (45.9)	62 (67.4)	
TARE, n (%)				0.796
No	99 (62.3)	40 (63.5)	59 (61.5)	
Yes	60 (37.7)	23 (36.5)	37 (38.5)	
AST, n (%)				0.002
<40 U/L	51 (32.1)	29 (46.0)	22 (22.9)	
≥40 U/L	108 (67.9)	34 (54.0)	74 (77.1)	
AFP, n (%)				0.005
<400 (ng/mL)	96 (62.0)	46 (75.4)	50 (53.2)	
≥400 (ng/mL)	59 (38.0)	15 (24.6)	44 (46.8)	
Combination treatment, n (%)				0.130
Sintilimab + Lenvatinib/Sorafenib/Bevacizumab	109 (68.5)	40 (63.5)	72 (75.0)	
Camrelizumab + Lenvatinib/Sorafenib	21 (13.2)	9 (14.3)	12 (12.5)	
Tislelizumab + Lenvatinib/Sorafenib	16 (10.1)	11(17.5)	5 (5.2)	
Cadonilimab + Lenvatinib	7 (4.4)	2 (3.2)	5 (5.2)	
Other medication regimens	6 (3.8)	1 (1.6)	2 (2.1)	
Prior treatment, n (%)				0.328
PTCD	9 (10.8)	6 (12.0)	3 (9.7)	
Curative treatment (Surgery, Ablation)	7 (8.4)	6 (12.0)	1 (3.2)	
Local-regional (TACE, HAIC, RFA)	54 (65.1)	28 (56.0)	24 (77.4)	
Target therapy (Sorafenib, lenvatinib)	10 (15.9)	7 (14.0)	3 (9.7)	
Radiotherapy	3 (4.8)	3 (6.0)	0 (0.0)	

Abbreviations: PVTT, portal vein tumor thrombosis; M, metastasis; ALBI, Albumin–Bilirubin; TARE, transarterial radioembolization; AST, aspartate aminotransferase; AFP, alpha-fetoprotein level; PTCD, percuteneous transhepatic cholangio drainage; HAIC, hepatic artery infusion chemotherapy; RFA, radiofrequency ablation.

**Table 2:** Univariate and multivariate logistic regression analyses of risk factors for early progression.

Variables	Univariate Analysis					Multivariate Analysis				
	β	S.E	Z	*p*	OR (95% CI)	β	S.E	Z	*p*	OR (95% CI)
ALBI grade										
1					1.00 (Reference)					
2	0.82	0.61	1.36	0.174	2.28 (0.69~7.48)					
3	1.29	0.65	1.99	0.047	3.63 (1.02~12.94)					
Tumor size										
<5 cm					1.00 (Reference)					
≥5 cm	0.89	0.34	2.62	0.009	2.44 (1.25~4.74)					
AST (U/L)										
<40 U/L					1.00 (Reference)					1.00 (Reference)
≥40 U/L	1.05	0.35	3.01	0.003	2.87 (1.44~5.70)	0.88	0.50	1.76	0.079	2.41 (0.90~6.43)
Alkaline phosphatase										
<150 U/L					1.00 (Reference)					
≥150 U/L	1.15	0.35	3.31	<0.001	3.15 (1.60~6.23)					
γ-glutamyl										
<50 U/L					1.00 (Reference)					1.00 (Reference)
≥50 U/L	1.31	0.37	3.50	<0.001	3.70 (1.78~7.71)	0.98	0.53	1.87	0.062	2.68 (0.95~7.51)
AFP (ng/mL)										
<400 (ng/mL)					1.00 (Reference)					
≥400 (ng/mL)	0.99	0.36	2.74	0.006	2.70 (1.33~5.49)					
Early AFP response										
No					1.00 (Reference)					1.00 (Reference)
Yes	−0.92	0.42	−2.20	0.028	0.40 (0.18~0.91)	−1.09	0.47	−2.34	0.019	0.34 (0.13~0.84)
WBC (10^9^/L)	0.13	0.07	1.98	0.047	1.14 (1.01~1.31)	0.12	0.08	1.47	0.140	1.13 (0.96~1.33)
Monocyte (10^9^/L)	1.70	0.77	2.21	0.027	5.45 (1.21~24.59)					

Abbreviations: ALBI, Albumin–Bilirubin; AST, aspartate aminotransferase; AFP, alpha-fetoprotein level; WBC, white blood cell; OR, Odds Ratio; CI, Confidence Interval.

**Table 3:** The relationship between early tumor response and early AFP response.

Treatment Response of 3 Months	Early AFP Response (n = 43)	Early AFP Nonresponse (n = 28)	*p* Value
CR	2 (4.6%)	1 (3.6%)	
PR	15 (34.9%)	2 (7.1%)	
SD	15 (34.9%)	6 (21.4%)	
PD + death	11 (25.6%)	19 (67.9%)	
ORR (CR + PR)	17 (39.5%)	3 (10.1%)	0.014
DCR (CR + PR + SD)	32 (74.4%)	9 (32.1%)	0.001

Abbreviations: AFP, alpha-fetoprotein level; CR, complete response; PR, partial response; SD, stable disease; PD, progressive disease; ORR, objective response rate; DCR, disease control rate.

## Data Availability

The data that support the findings of this study are available from the corresponding author upon reasonable request.

## Abbreviation

Abbreviation	Full form
ICI	immune checkpoint inhibitor
OS	overall survival
mOS	median overall survival
AFP	alpha-fetoprotein
AUC	area under curve
HCC	hepatocellular carcinoma
PFS	progression-free survival
mPFS	median progression-free survival
HBV	hepatitis B virus
BCLC	Barcelona Clinic Liver Cancer
ALBI	Albumin–Bilirubin
CRP	C-reactive protein
NLR	neutrophil–lymphocyte ratio
ECOG PS	Eastern Cooperative Oncology Group Performance Status
SD	standard deviation
IQR	interquartile range
CI	confidence interval
ROC	receiver operating characteristic
OR	odds ratio
DCA	decision curve analysis
KM	Kaplan–Meier
PVTT	portal vein tumor thrombosis
M	metastas
TARE	transarterial radioembolization
AST	aspartate aminotransferase
ALP	alkaline phosphatase
PTCD	percuteneous transhepatic cholangio drainage
HAIC	hepatic artery infusion chemotherapy
RFA	radiofrequency ablation
WBC	white blood cell
CR	complete response
PR	partial response
SD	stable disease
PD	progressive disease
ORR	objective response rate
DCR	disease control rate
